# A Review on Noma: A Recent Update

**DOI:** 10.5539/gjhs.v8n4p53

**Published:** 2015-07-30

**Authors:** Nipun Ashok, Bassel Tarakji, Shorouk Darwish, Jean C. Rodrigues, Mohammad A. Altamimi

**Affiliations:** 1 Department of Oral and maxillofacial sciences, Alfarabi College of dentistry and nursing, Riyadh, Saudi Arabia; 2 Department of Restorative dentistry, Alfarabi College of dentistry and nursing, Riyadh, Saudi Arabia

**Keywords:** cancrum oris, gangrene, noma, pathogenesis

## Abstract

Noma is a gangrenous infection primarily affecting under developed countries. The aim of this paper was to review all recent articles on noma from January 2003 to August 2014 and briefly update the latest information related to the topic. A literature search was done on PUBMED using the keywords “noma / cancrum oris”. Noma is commonly seen in malnourished children. There has been an increased incidence of noma in HIV patients. Apart from these, noma has also been reported in association with cyclic neutropenia, herpetic stomatitis, leukemia, Down's syndrome and Burkett's disease. Treatment of acute noma includes transfusion of blood and intravenous fluids, administration of antibiotics, putting the patient on a high protein diet and debridement of necrotic areas. Surgical phase is usually initiated 6 to 18 months after a period of quiescence. Although, the mortality rate associated with noma has reduced significantly with the advent of modern generation antibiotics, the functional, cosmetic and psychological challenges associated with the destruction of soft or hard tissues still remains a huge challenge. Adequate steps must be implemented by the government or medical professionals to prevent the disease and provide an early intervention.

## 1. Introduction

Noma is a “gangrenous affection of the mouth, especially attacking children in whom the constitution is altered by bad hygiene and serious illness especially from eruptive fevers, beginning as an ulcer of the mucous membrane with edema of the face extending from within out, rapidly destroying the soft tissues and the bone and almost always quickly fatal”. The term noma originates from the greek word “nomein” which means to devour or to graze (Auluck & Pai, 2005).

## 2. Methods

Although the incidence of noma is very high in undeveloped countries and the consequence very severe, it has not received much attention. The aim of this paper was to review the recent articles on noma and update the latest information regarding noma. A literature search was done for articles on PUBMED using the keywords noma, cancrum oris from January 2003 till August 2014. A total of 44 articles were included in the review, which gave an insight to the history, epidemiology, risk factors, pathogenesis, microbiology, complications and management of noma.

## 3. History

Noma was described in ancient Greek and roman medical texts and was found to be common by medieval and renaissance European writers (Vinetz, 2008). Noma was first reported by Hippocrates in 5^th^ century B.C (Baratti-Mayer et al., 2003). In the 19^th^ century, A.L Richter reported the presence of noma throughout Europe and he associated this disease with malnutrition and childhood infection such as measles. Noma had almost disappeared from Europe and North America by the end of the 19^th^ century, except for the cases seen in concentration camps during the Second World War, but there has been an increasing incidence of this disease in underdeveloped countries. In 1994, the World Health Organization (WHO) described noma as a health priority and an action program was initiated involving the WHO, the United States National institute of health and the University of Maryland, Baltimore (K. Ogbureke & E. Ogbureke, 2010).

## 4. Epidemiology and Risk Factors

The annual incidence and prevalence of noma was reported to be 1,40,000 and 7,70,000 cases respectively (Enwonwu, 2006; Tonna, Lewin, & Mensh, 2010). Finding the exact incidence and prevalence of noma was difficult because of the high mortality rate, incidence in underdeveloped countries, difficulty in registering, controlling and following up because of the affected people being nomadic and the isolation of patients because of the social stigma associated with noma (Baratti-Mayer et al., 2003).

Most cases of noma are reported from the so-called noma belt, which is located south of the Sahara and runs across Africa from Senegal to Ethiopia (Marck, 2013). Recently, isolated cases of noma have been reported from developed countries (Chiandussi, Luzzati, Tirelli, Di Lenarda, & Biasotto, 2009). Noma is commonly seen in a population with extreme poverty, severe malnutrition, unsafe drinking water, poor sanitation, poor oral health practices, high infant mortality, limited access to high quality health care and intrauterine growth retardation (Tonna et al., 2010). Recently, an increased incidence of noma has been reported in patients with Human Immune deficiency Virus (HIV) infection (Baratti-Mayer et al., 2003; Marck, 2013). Cases of noma reported in HIV patients are shown in Table 1. Noma was also reported in patients with cyclic neutropenia (Erbagci, 2003), leukemia (Santos, Neri, & Chiattone, 2011), Down's syndrome (Lembo, De Leonibus, Francia, Lembo, & Ayala, 2011), Burkett's disease (Millogo, Konsem, Ouedraogo, Ouoba, & Zwetyenga, 2012) and herpetic stomatitis (Fasola, Obiechina, & Arotiba, 2003).

**Table 1:** Noma cases reported in HIV patients

Authors (year)	Number of cases
Faye et al. (2003)	2
Ondzotto, Ibara, Mowondabeka, & Galiba (2004)	4
Naidoo & Chikte (2004)	1
Chidzonga & Mahomva (2008)	48
Chidzonga & Mahomva (2008)	1
Diallo, Camara, Bah, Barry, & Cisse (2009)	5
Pacheco-Tenza, Hernandez-Ros, Gregori-Colome, & Navarro-Lopez V (2010)	1
Koech KJ (2010)	1
Millogo, Konsem, Ouedraogo, Ouoba, & Zwetyenga (2012)	14
Lubala, Mutombo, Mukuku, Ilunga, & Shongoya (2012)	1
Masipa et al. (2013)	2
Van niekerk, Khammissa, Altini, Lemmer, & Feller (2014)	1

Malnutrition is considered to be an important risk factor for noma (Baratti-Mayer et al., 2003). In Africa, most of the cases were reported during the dry season when food is scarce and when incidence of measles is highest (Enwonwu, Falkler, & Philips, 2006). Debilitating diseases like malaria and measles were considered to be a significant risk factors or precursors to noma. Measles could be an important risk factor because of the associated immunosuppression (Baratti-Mayer et al., 2003).

A recently conducted prospective study by Baratti-Mayer et al. (2013) concluded the predictors of noma to be severe malnutrition, recent respiratory or diarrhoeal syndrome, the number of previous pregnancies in the mother, an altered oral microbiota when compared with controls and the absence of chickens at home. According to Adeola and Obiadazie (2009), some cases in northern Nigeria resulted due to the lack of maternal care, where mothers leave their babies in the care of their grandmother as soon as they are delivered, as they have to compete with their mates for the husband. Grandmothers are less educated regarding a balanced diet leading to malnourishment in babies and resulting in noma.

## 5. Pathogenesis

The exact etiology of noma is not known but it is believed to be multifactorial in nature. Researchers initially believed that bone exposure caused by acute necrotizing ulcerative gingivitis (ANUG) could act as a passage point for noma. But the present consensus is that ANUG is a precursor for noma (Baratti-Mayer et al., 2003).

It is suggested that factors such as malnutrition, weakened immune functions and prior viral infection, all worsened by poor oral hygiene could lead to reduction in host resistance and favor the development of oral ulcers. These lesions can serve as entry sites for microorganisms responsible for the disease process (Huyghe et al., 2013). When poverty prevails, there appears to be a synergistic relation between malnutrition, weakened immune functions and increased susceptibility to infections. In malnourished subjects, IgA, an important component of the mucosal immune system, is significantly reduced whereas plasma concentration of pro-inflammatory cytokines and C-reactive protein is increased when compared to healthy counterparts. Infections such as AIDS, measles and malaria could also lead to a shift from pro-inflammatory cytokines to anti-inflammatory cytokines (Enwonwu et al., 2006).

Rapid destruction of hard and soft tissues seen in noma could be attributed to immunopathological response to microbial factors rather than microbiological factors alone. Enwonwu, Phillips and Savage (2005) found higher plasma levels of anti-inflammatory and pro-inflammatory cytokines in children with necrotizing ulcerative gingivitis when compared to controls. Research has shown that oral epithelial cells and other resident cells secrete several pro-inflammatory cytokines and chemokines in response to bacterial products which in turn can stimulate the expression of matrix metalloproteinase. This matrix metalloproteinase can cause destruction of both hard and soft tissues (Enwonwu et al., 2006).

## 6. Microbiology

Noma being an opportunistic infection, the role of specific microorganisms in the pathogenesis has not been explained. This is because of a large range of uncultivable microorganisms, the disease usually develops rapidly in remote geographic areas making it difficult for early microbiological analysis and its prevalence in populations whose normal oral flora is poorly investigated (Huyghe et al., 2013). Recent advancements in microbial techniques have helped in better identification and characterization of microorganisms seen in noma.

A number of potential pathogens were found in abundance in the sites of noma which include *Prevotella* *melaninogenica, Corynebacterium pyogenes, Fusobacterium nucleatum, Bacteroides fragilis*, *Bacillus cereus*, *Prevotella intermedia* and *Fusobacterium necrophorum* (Baratti-Mayer et al., 2003). Microbial analysis in the early 20^th^ century revealed the presence of spirilliform and fusiform microorganisms in biopsy samples taken from the transitional zone between gangrenous and healthy tissues (Marck, 2013). Later studies reported that *Fusobacterium necrophorum*, a predominant animal pathogen to be the most common microorganism isolated from the disease sites in Nigerian children. It was suggested that *Fusobacterium necrophorum* could be a trigger organism for noma. This microorganism produces various toxins and has been associated with necrotizing infections in animals and it may contaminate livestock and potentially infect children (Enwonwu et al., 2006; Baratti-Mayer et al., 2013).

A recent study conducted by Hughye et al. (2013) contradicted the involvement of *Fusobacterium necrophorum* as an etiologic agent. Known periodontal pathogens like *aggregatibacter actinomycetemcomitans, capnocytophaga, porphyromonia* and *fusobacteria* were more prevalent in healthy samples compared to those with noma. Studies by Hughye et al. (2013) and Bolivar et al. (2012) identified *prevotella intermedia* and *peptostreptococus* to be more clearly associated with noma.

## 7. Clinical Features

Systemic manifestations of noma include fever, tachycardia, lymphadenopathy, high respiratory rate, anorexia, general edema and ascites. Medical history reveals a parasitic or viral infection (measles, malaria) in the recent past, recurrent fever and diarrhea. Blood examination reveal a low hemoglobin concentration and white blood cell count, elevated erythrocyte sedimentation rate and hypoalbuminaemia (Auluck & Pai, 2005; Enwonwu et al., 2006; Baratti-Mayer et al., 2013). Fresh cases of noma are seen primarily in the 1 to 4 age group. Children with acute noma suffer from linear growth retardation and are severely affected (Enwonwu, Philips, & Ferrell, 2005; De Onis & Blosner, 2003). The course of noma is very rapid and death can occur in some days. HIV patients reported with noma may have a very low CD4 count (Masipa et al., 2013).

It is believed that noma is an extension of necrotizing ulcerative gingivitis (NUG) which is characterized by gingival edema, necrosis, bleeding and pain (Enwonwu et al., 2006). NUG may progress to necrotizing periodontitis (NP) and later to necrotizing stomatitis. In necrotizing stomatitis, NUG or NP spread beyond the mucogingival junction to affect the alveolar, buccal, lingual or palatal mucosa. Untreated necrotizing stomatitis may progress to noma, but some cases of noma are reported without a previous history of necrotizing stomatitits (Masipa et al., 2013).

The first recognized sign of noma is edema of cheek, or gingiva or both. A greyish black area appears on the external surface of the cheek opposite to the intraoral lesion within the next few days, which later on becomes a well-defined black necrotic zone. This necrotic zone acquires a cone shape and rapidly sloughs away (Enwonwu et al., 2006). Intra-oral manifestations include sequestration of the exposed bone and teeth, halitosis, pseudomembranes, excessive salivation, spontaneous gingival bleeding and loss of tips of interdental gingival papilla. Sometimes the necrosis is very severe that both maxilla and mandible are completely destroyed extending up till the nose, upper lip, pre-maxilla and the infraorbital margin (Baratti-Mayer et al., 2003; Enwonwu et al., 2006; Tonna et al., 2010; Baratti-Mayer et al., 2013).

## 8. Differential Diagnosis

Differential diagnosis for noma includes leprosy, leishmaniasis, post kala-azar dermal leishmaniasis, oral cancer, clostridial or streptococcal gangrene and Stewart's granuloma (Tonna et al., 2010). Neonatal noma is a rare clinical syndrome with gangrene of orofacial tissues involving both term and preterm infants in the first week of life and resembles noma in older children (Auluck & Pai, 2005). The predisposing factors for noma neonatorum are considered to be preterm birth weight and severe intrauterine growth retardation (Enwonwu, 2006). Some authors believe that neonatal noma is a distinct identity, where neonates are affected with pseudomonas sepsis (Baratti-Mayer et al., 2003; Vaidyanathan, Tullu, Lahiri, & Deshmukh, 2005).

## 9. Complications

Mortality used to be a common complication of noma. With the use of modern antibiotics and better nutrition, mortality rate has reduced from 90% to 8-10% (Auluck & Pai, 2005; Marck, 2003). Noma can result in trismus, sequestration of jaws, fibrous ankylosis of temporomandibular joint, oro-nasal fistula, damage to permanent tooth bud, early loss of decidious teeth and hypoplasia of maxilla or mandible. Most of the noma patients have difficulty in mastication because of loss of soft and hard tissue. Severe cosmetic disfigurement can also take place from the resulting scarring and loss of tissue (Holle, 2009; Jayasankar, Chavda, & Shah, 2004; Woon, Sng, Tan, & Lee, 2010). According to Yunusa and Obembe (2012), patients reported of a high psychiatric morbidity after noma.

## 10. Management and Prevention

Acute noma is managed by blood transfusion, transfusion with intravenous fluid for correction of dehydration and electrolyte imbalances, treatment of associated diseases like malaria and measles, the administration of antibiotics and putting the patient on a high protein diet (Auluck & Pai, 2005; Enwonwu, 2006). The affected area has to be debrided with dilute hydrogen peroxide or Edinburgh university solution of lime (EUSOL) or saline and any remaining tissue slough and sequestrate has to be removed. A course of antibiotics (ampicillin-cloxacillin and metronidazole), multivitamin preparation and antiseptic therapy has to be administered for a week. Patients are advised to rinse their mouth with chlorhexidine gluconate (0.12-0.2%) daily. The patient has to be screened for HIV infection and referred appropriately (Baratti-Mayer et al., 2003; Enwonwu et al., 2006; Baratti-Mayer et al., 2013).

Surgical correction is initiated after a period of disease quiescence of at least 6 to 18 months. The aim of the surgical procedure is to restore oral speech, oral competence and acceptable aesthetics. Reconstructive surgery in children is delayed till the patient matures because it allows the defect to contract and reduce in size, allows sufficient cooperation postoperatively and ensures adequate tissue for reconstruction. Trismus which most often results from extra-articular ankyloses (fibrosis) is corrected by complete excision of fibrosis followed by physiotherapy (Woon et al., 2010; Baratti-Mayer et al., 2013). Closure of tissue defects is usually done by local, pedicled or free flaps. Various techniques employed include prefabricated scapular flaps, free radial forearm flap, anterolateral thigh flap, pedicled supraclavicular flap, waltzing flap and Gillies fan flap (Woon et al., 2010; Hartman, Van Damme, Sauter, & Suominen, 2006; Giessler et al., 2007; Vinzenz, Holle, & Wuringer, 2008; Hartman, Van Damme, Rayatt, & Kuokkanen, 2010; Bello, 2012). An analysis by Bouman et al. (2010), found early success rate of 59% after surgical treatment of noma, but the success rate was significantly reduced after complex surgical procedures.

Measures needed to prevent noma include administration of nutritious food, exclusive breast feeding during the first three to six months of life, inculcation of proper oral hygiene practices, immunization against endemic diseases like measles, segregation of animals from human living areas and creating a proper awareness about noma (Enwonwu, 2006).

## 11. Conclusion

Noma is a debilitating disease primarily affecting the poor and has been called the “face of poverty”. It occurs among the underprivileged society where patients do not have access to good medical care. Noma can be prevented to a large extent by providing good nutrition and water facilities, vaccinations and by maintaining good hygiene. The government and health organizations need to take adequate steps to improve the social living conditions of individuals living in noma susceptible areas. Efforts should be made by medical professionals to provide early intervention and medical care to the patients so that mortality can be reduced and tissue destruction can be minimized.
